# Micronutrient deficiencies and anemia among adolescents in rural south India: A game changer for public health interventions

**DOI:** 10.6026/973206300210347

**Published:** 2025-03-31

**Authors:** Ravishankar Suryanarayana, Harish Rangareddy, Shilpa M.D, Bindu Madhavi R., Karthik S.

**Affiliations:** 1 Department of Community Medicine, Sri Devaraj Urs Medical College constituent college of Sri Devaraj Urs Academy of Higher Education and Research, Kolar, Karnataka, India; 2 Department of Biochemistry, Haveri Institute of Medical Sciences, Haveri, Karnataka, India; 3 Department of Pathology, Sri Devaraj Urs Medical College constituent college of Sri Devaraj Urs Academy of Higher Education and Research, Kolar, Karnataka, India; 4 Department of Microbiology, Sri Madhusudan Sai Institute of Medical Sciences and Research, Chikkaballapur, Karnataka, India; 5 Department of Pediatrics, Sri Devaraj Urs Medical College constituent college of Sri Devaraj Urs Academy of Higher Education and Research, Kolar, Karnataka, India

**Keywords:** Public health, school age population, micronutrients, anemia

## Abstract

Anemia remains a significant adolescent health concern with multifactorial causes. Hence, 253 adolescents were assessed for anemia using biochemical markers. Anemia was detected in 17.4% (95% CI: 6.3-28.5%) and it is more frequent among girls (20.16%) than boys (14.7%). It correlated with faith (p=0.025) but not with socio-economic status, caste, or parental education. Anemic participants had lower serum iron (p=0.001) and higher TIBC (p=0.025) suggesting iron deficiency. Ferritin was markedly lower in anemic boys compared to anemic girls. Elevated TIBC in 78.26% reinforced the prevalence of iron deficiency. These findings highlight the need for targeted nutritional interventions and screening programs.

## Background:

Micronutrient deficiency is a major public health concern globally, with adolescents being one of the most susceptible groups [1]. In recent years, the global conversation around adolescent health has increasingly focused on the critical role of nutrition in shaping physical and cognitive development. Among adolescents aged 10-19 years, micronutrient deficiencies stand out as a significant factor, particularly in the context of anemia [2]. Inadequate nutrition, along with deficiencies in micronutrients leading to iron deficiency anemia, continues to be a major factor contributing to disability and mortality among adolescents [3]. Iron deficiency can primarily be attributed to inadequate dietary intake of bioavailable iron. Among adolescents, low iron status can result from menstrual blood loss in females and prolonged bleeding, which negatively impacts growth during adolescence when red blood cell mass is expanding [1]. Anemia, one of the most reported consequences of iron deficiency, is characterized by a reduction in the blood's oxygen-carrying capacity, leading to symptoms such as fatigue, weakness and reduced work capacity [4]. In developing countries, anemia in adolescence affects cognitive performance, behavioural characteristics and physical growth. It also adversely affects immunity and increases morbidity from infections [5]. Anemia among adolescents is a common phenomenon due to limited access to fruits, vegetables and animal-source foods. Poor socioeconomic status further contributes to inadequate micronutrient intake, particularly among rural children [6]. As the academic performance of adolescents is a prime determinant of their career opportunities and future economic prospects, ensuring proper nutrition is a crucial investment in their future by improving their learning capabilities [7]. Therefore, it is of interest to evaluate key micronutrient levels-serum iron, ferritin, TIBC, transferrin and folic acid-and their association with dietary habits.

## Methodology:

## Study design:

A descriptive cross-sectional study was conducted from December 2022 to February 2023, following approval from the Central Ethics Committee (CEC No. SDUAHER/Res.Proj/171/2020-21). The sample size was determined using a 20.7% prevalence of anemia among adolescents, based on a prior study in rural Karnataka [8]. With a 95% confidence interval and a 5% absolute error, the estimated sample size was 250 students. A list of government high schools and higher primary schools in rural Kolar was obtained from the Educational Officer. Among 22 schools, four were randomly selected using simple random sampling. From 460 students across grades 6 to 10, approximately 50 students from five randomly chosen clusters were enrolled through cluster sampling. Data collection involved a pretested questionnaire assessing socio-demographic details and anthropometric measurements. Venous blood samples (5 mL) were collected to analyze haemoglobin levels, with anemia classified according to WHO criteria: mild (10-11.9 g/dL), moderate (7-9.9 g/dL) and severe (<7 g/dL). Biochemical investigations included serum iron (two-point rate method), transferrin (c), total iron-binding capacity (TIBC, iron-binding dye method) and ferritin. Additional tests comprised folic acid, vitamin B12, complete blood count (CBC), peripheral blood smear and stool examination. The existing government-led Weekly Iron and Folic Acid Supplementation (WIFS) program for adolescents with anemia was also documented.

## Statistical analysis:

Collected data were coded into an Excel spread sheet and normality was assessed using skewness and statistical plots. Quantitative biochemical parameters, including transferrin, TIBC, ferritin, iron and folic acid, were expressed as mean ± standard deviation (SD), while categorical variables were summarized as frequencies and percentages. Group comparisons were conducted using the independent t-test and ANOVA for continuous variables and the chi-square test for categorical variables. Multiple linear regressions were performed to evaluate the association between hemoglobin levels and micronutrient deficiencies. Statistical significance was determined based on p-values, with appropriate thresholds applied for interpretation.

## Results:

## Prevalence and classification of anemia:

The prevalence of anemia in the study population was 17.4% (95% CI: 6.3-28.5%). Among boys, the prevalence was 14.7% (95% CI: 8.59-20.81%), whereas among adolescent girls, it was 20.16% (95% CI: 13.1-27.22%). The mean Hb levels among girls and boys were 12.67 ± 1.34 g/dL and 13.04 ± 1.48 g/dL, respectively. (Table 1) presents the distribution of anemia severity across various demographic characteristics. Among the participants, anemia was more prevalent in females (25.2%) compared to males (14.7%), though the association was not statistically significant (χ^2^ = 2.784, p = 0.248). The prevalence of anemia varied across age groups, with higher proportions observed among younger adolescents, but no statistically significant trend was noted (χ^2^ = 5.44, p = 0.065). A significant association was found between anemia and religion (χ^2^ = 4.990, p = 0.025), with Hindu students showing a higher proportion of mild and moderate anemia compared to Muslim students. However, no significant associations were observed with caste (χ^2^ = 1.643, p = 0.440), socioeconomic status (χ^2^ = 0.183, p = 0.669), or type of family (χ^2^ = 0.359, p = 0.836). Parental education levels did not show a statistically significant relationship with anemia, though a higher prevalence was noted among students whose parents had lower educational attainment. Similarly, anemia was more common among participants with more than two siblings, but this association was not statistically significant (χ^2^ = 2.370, p = 0.126). These findings highlight the complex interplay of socio-demographic factors in influencing micronutrient deficiencies, necessitating targeted interventions focusing on at-risk groups.

## Biochemical parameters in anemic and non-anemic adolescents:

Table 2 summarizes the biochemical parameters among adolescents with and without anemia. The mean serum iron levels were significantly lower in anemic adolescents (n=44) (73.70 ± 40.82 µg/dL) compared to non-anemic counterparts (n=209) (95.53 ± 41.41 µg/dL, p = 0.001). Total iron-binding capacity (TIBC) was significantly elevated in anemic individuals (433.02 ± 82.75 µg/dL vs. 400.67 ± 88.84 µg/dL, p = 0.025), suggesting increased iron mobilization in response to iron deficiency. Transferrin, folic acid and ferritin levels were slightly lower in anemic individuals, but these differences did not reach statistical significance.

## Ferritin and iron deficiency:

Ferritin levels were significantly lower in anemic adolescent boys 61% (n=51) and 39% (n=23) girls with p = 0.00024 as shown in (Table 3). However, the distribution of transferrin levels did not differ significantly between genders (p = 0.156). Iron deficiency was more prevalent among boys (19.5%) compared to girls (12.8%), further indicating potential dietary or physiological differences influencing iron metabolism as shown in Table 4.

## Total iron binding capacity (TIBC):

Table 5 presents the distribution of total iron-binding capacity (TIBC) levels among boys and girls. The majority of adolescents (78.26%) had TIBC levels exceeding 497µg/dL, indicating a high prevalence of increased iron-binding capacity, which may reflect iron deficiency. Among these individuals, boys constituted a slightly higher proportion (54.54%) compared to girls (45.46%). Conversely, 11.47% of participants exhibited TIBC levels below 265µg/dL, suggesting a lower iron-binding capacity, which could be indicative of conditions such as iron overload or inflammation. Notably, a higher proportion of girls (65.52%) fell into this category compared to boys (34.48%). The normal TIBC range (265-497 µg/dL) was observed in only 10.27% of the study population, with girls (57.69%) being slightly more represented than boys (42.31%). This suggests that the majority of adolescents in the study exhibited deviations from the normal range, primarily showing elevated TIBC levels, which may point toward an increased demand for iron due to suboptimal iron stores. The predominance of elevated TIBC levels suggests a high likelihood of iron deficiency among adolescents in the study population. Given the physiological iron demands during adolescence, these findings underscore the need for dietary interventions and screening programs to address potential iron insufficiency.

## Folic acid levels and diet:

Table 6 presents the distribution of folic acid levels among boys and girls in the study population. Overall, there was no statistically significant association between folic acid levels and gender (p = 0.052), although the borderline significance suggests a potential trend that warrants further investigation. The vast majority of adolescents (94.1%) had folic acid levels within the normal range (2.76-17 ng/mL), indicating sufficient dietary intake of folate-rich foods such as leafy vegetables, legumes and fortified cereals. Notably, 5.9% of participants exhibited folic acid deficiency (<2.76 ng/mL), with a higher prevalence among girls (73.4%) compared to boys (26.6%). This disparity may reflect differences in dietary habits, increased physiological demands, or variations in nutrient absorption.

The high proportion of adolescents with normal folic acid levels suggests an overall adequate folate intake in the study population. However, the presence of folic acid deficiency, particularly among girls, highlights the importance of targeted nutritional interventions to prevent potential complications associated with folate insufficiency, such as anemia and impaired cognitive development. Further studies examining dietary patterns and socioeconomic factors influencing folic acid status could provide deeper insights into these findings.

## Regression analysis of hemoglobin levels:

A linear regression model demonstrated that 12.3% of the variation in hemoglobin levels could be attributed to biochemical parameters as depicted in Table 7. Serum iron levels showed a strong positive association with hemoglobin (p<0.001), while TIBC was negatively associated with hemoglobin levels (p=0.020), indicating a potential compensatory response to iron depletion.

## Dietary intake patterns among adolescents:

The dietary habits of adolescents play a crucial role in determining their micronutrient status, particularly folic acid levels. Table 8 presents the consumption patterns of key folate-rich food groups, including leafy vegetables, fruits and pulses/sprouts, among boys and girls in the study population. A high proportion of adolescents reported consuming leafy vegetables, with 94.6% of boys and 95.2% of girls including them in their diet. Similarly, fruit consumption was prevalent among 95.3% of boys and 91.1% of girls. However, the intake of pulses and sprouts was comparatively lower, with 72.1% of boys and only 58.9% of girls reporting regular consumption. The high intake of leafy vegetables and fruits suggests an overall good dietary pattern supporting adequate folic acid levels in the majority of adolescents. However, the relatively lower consumption of pulses and sprouts, especially among girls, may contribute to micronutrient gaps and potential deficiencies. These findings underscore emphasize the importance of dietary iron intake and suggest that higher iron availability correlates with better haematological outcomes, while elevated TIBC may indicate underlying iron deficiency. There is a need for targeted nutritional education to encourage a more balanced intake of folate-rich foods, particularly among female adolescents, to support optimal growth and development.

## Discussion:

In the present study, the prevalence of anemia was 17.4%. Among boys, the prevalence was 14.7%, while among adolescent girls, it was 20.16%, which is lower compared to NFHS survey estimates. The National Family Health Survey indicated that the prevalence of anemia among Indian adolescents aged 15-19 years was 59.1% in girls and 31.1% in boys [9]. In a study by Mahajan *et al.* anemia among late adolescents in tribal populations showed that girls aged 14 to 18 years had a significantly higher prevalence of anemia compared to boys [10]. The overall mean hemoglobin level was 13.04 ± 1.48 g/dL. The mean hemoglobin level among girls was 12.67 ± 1.34 g/dL, while among boys; it was 13.04 ± 1.48 g/dL. Anemia was comparatively higher among early adolescents (11-14 years) at 59.1%, whereas it was 40.9% among late adolescents (15-17 years). A cross-sectional secondary data analysis by Chauhan *et al.* observed that the prevalence of anemia in early adolescent boys (10-14 years) was 33.6%, while among girls, it was 55.2%. Among late adolescents (15-19 years), the prevalence was 30.6% in boys and 65.3% in girls [11]. A cross-sectional study by Shettar *et al.* also reported similar observations [12]. The prevalence of anemia was higher among adolescents belonging to joint or joint-extended families. The study also found that anemia prevalence was lower among adolescents whose mothers had an education level of matriculation or above. However, there was no statistically significant association between anemia and paternal education. Anemia was more predominant among families with fewer than two siblings. However, except for religion, none of the demographic factors-including age, caste, gender, parental education, number of siblings, family type and socioeconomic status-were significantly associated with anemia. In a similar cross-sectional study by Verma *et al.* anemia was found to have a significant association with socioeconomic status and parental education. However, factors such as age at menarche, age, family size and type of family were not associated with anemia [13]. Similarly, Goyal *et al.* reported that factors such as place of residence, type of school, birth order, type of family and mother's occupation were significantly associated with anemia [14]. A cross-sectional study by Mamokem *et al.* made a similar observation, reporting a highly significant difference in mean serum iron levels between anemic and non-anemic adolescent schoolchildren (p<0.001) [15]. One of the findings in this study was the significant increase in TIBC levels among adolescents with anemia (p=0.025). Mamokem *et al.* also reported a significant increase in TIBC levels among iron-deficient anemic individuals compared to the non-anemic group (p=0.001) [15].

In this study, 12.6% of schoolchildren had transferrin levels below normal limits. The analysis of transferrin levels among vegetarians, non-vegetarians and egg-eaters showed no significant difference in transferrin levels between boys and girls within each dietary group. However, boys had better transferrin levels compared to girls, regardless of diet. In the present study, 26.12% of anemic children, across different grades of anemia, had ferritin deficiency. Although the difference in mean ferritin levels between anemic groups was not strong, low ferritin levels could still be considered a contributing factor to anemia. The difference in mean ferritin levels between anemic and non-anemic groups was significant (p=0.046), with ferritin levels decreasing as anemia severity increased. Mamokem *et al.* also reported a highly significant difference in mean serum ferritin levels between iron-deficient anemic and non-anemic adolescent schoolchildren [15]. The difference in ferritin levels between boys and girls was significant (p=0.00024). A cross-sectional study by Andriastuti *et al.* in suburban schools among children aged 6-18 years also reported a marginally significant reduction in serum ferritin levels among girls compared to boys (p=0.073) [16]. In a study by Srinath *et al.* anaemia prevalence was 60.7%. Of 260 participants tested for micronutrient deficiencies, 65.4% had ferritin deficiency, 18.5% had folate deficiency and 47.7% had vitamin B12 deficiency and additionally, 98.2% of participants had inadequate dietary diversity [17].

Among adolescents with anemia, there was a significant increase in TIBC levels (p=0.006**). In anemic adolescents, the blood's capacity to bind with iron and transport it throughout the body was reduced. This suggests that the body was not efficiently utilizing or distributing iron, which is essential for hemoglobin production-a key component of red blood cells responsible for oxygen transport. As a result, the diminished ability to bind and transport iron could exacerbate anemia symptoms, leading to fatigue, weakness and other health issues due to inadequate oxygen supply to tissues. Iron and folic acid are crucial nutritional requirements for adolescent girls. Folic acid, in particular, helps prevent folate deficiency, which is implicated in anemia. In this study, boys had marginally higher folic acid levels (5.14 ± 2.09) compared to girls (4.95 ± 2.34), but the difference was not statistically significant. Leafy vegetables such as spinach, kale and broccoli are rich sources of iron and calcium. In this study, boys consumed leafy vegetables more frequently than girls and those who consumed them had higher iron levels. Since the study participants were from rural areas, the consumption of leafy vegetables was relatively common. Ghatpande *et al.* observed that adolescent girls who consumed green leafy vegetables, such as amaranth, had significantly higher iron and ferritin levels compared to non-consumers [18]. A balanced diet rich in folate-containing foods is essential for maintaining adequate folic acid levels. In this study, 94% of adolescents had normal folic acid levels, indicating adequate consumption of green leafy vegetables, sprouts and fruits. Apriningsih *et al.* found a significant association between regular iron-folic acid consumption and lower anemia prevalence among adolescent girls [19].

## Conclusion:

Inadequate intake of essential nutrients contributes significantly to deficiencies among adolescents. However, large scale data to inform targeted interventions remain limited. Understanding dietary patterns, underutilization of local foods and their role in micronutrient deficiencies is crucial. Further, promoting balanced dietary practices and supplementation programs could help alleviate micronutrient deficiencies and reduce the burden of anemia in this population.

## Figures and Tables

**Table 1:** Classification of anemia based on demographic characteristics

**Demographic Variables**	**Moderate Anemia (7-9.9 g/dL)**	**Mild Anemia (10-11.9 g/dL)**	**Normal (>12 g/dL)**	**χ^2^ Value**	**P-Value**
**Age (years)**					
11	0	3	20		
12	0	6	36		
13	1	11	53		
14	2	3	28	5.44	0.065
15	5	6	51		
16	2	5	20		
17	0	0	1		
**Gender**					
Girls	8	17	99	2.784	0.248
Boys	3	16	110		
**Religion**					
Hindu	10	31	205	4.99	0.025*
Muslim	3	0	4		
**Caste**					
General	1	0	5		
Other Backward Classes	1	13	88	1.643	0.44
Scheduled Castes/ Scheduled Tribes	9	20	116		
Socioeconomic Status					
Above Poverty Line (APL)	1	1	13		
Below Poverty Line (BPL)	10	32	196	0.183	0.669
**Type of Family**					
Nuclear	3	13	78		
Joint	7	15	109	0.359	0.836
Joint Extended	0	6	22		
**Education of Mother**					
Illiterate	5	11	57		
Primary/Higher Primary/High School	4	14	81	2.25	0.284
Matriculation/PUC/Degree	3	7	71		
**Education of Father**					
Illiterate	5	6	43		
Primary/Higher Primary/High School	1	15	57	2.708	0.258
Matriculation and above	5	12	109		
**Siblings**					
Less than 2	9	27	188	2.37	0.126
More than 2	4	4	21		
*p< 0.05 indicates statistical significance.

**Table 2:** Biochemical parameters in anemic and non-anemic adolescents

**Parameter**	**Normal (Mean ± SD) (n=209)**	**Anemic (Mean ± SD) (n=44)**	**p value**
Iron (µg/dL)	95.53 ± 41.41	73.70 ± 40.82	0.001*
Transferrin (mg/dL)	285.60 ± 69.97	285.20 ± 65.79	0.547
TIBC (µg/dL)	400.67 ± 88.84	433.02 ± 82.75	0.025*
Ferritin (mg/mL)	29.17 ± 16.34	30.05 ± 46.53	0.561
Folic Acid (ng/mL)	5.12 ± 2.31	4.71 ± 1.74	0.396
*p< 0.05 indicates statistical significance.

**Table 3:** Ferritin classification in boys and girls

**Ferritin levels (mg/mL)**	**Boys (n, %)**	**Girls (n, %)**	**Total (n, %)**	**χ^2^ Value**	**p value**
<24	51 (60.9)	23 (39.1)	74 (29.3)	13.46	0.00024*
24-336	78 (43.6)	101 (56.4)	179 (70.7)		
Total	129	124	253		
*p< 0.05 indicates statistical significance.

**Table 4:** Categorization of students by serum iron levels

**Iron Levels (µg/dL)**	**Boys (n, %)**	**Girls (n, %)**
<49 (boys) / <37 (girls)	25 (19.5)	16 (12.8)
49-181 (boys) / 37-170 (girls)	104 (80.5)	107 (85.6)
>181 (boys) / >170 (girls)	0 (0.0)	1 (1.6)

**Table 5:** Distribution of TIBC levels in adolescents

**TIBC Levels (µg/dL)**	**Boys (n, %)**	**Girls (n, %)**	**Total (%)**
265-497 (Normal)	11 (42.31%)	15 (57.69%)	26 (10.27%)
> 497	108 (54.54%)	90 (45.46%)	198 (78.26%)
< 265	10 (34.48%)	19 (65.52%)	29 (11.47%)
Total	129	124	253

**Table 6:** Folic acid levels in adolescents

**Folic Acid Levels (ng/mL)**	**Boys (n, %)**	**Girls (n, %)**	**Total (%)**	**χ^2^**	**p value**
< 2.76	4 (26.6%)	11 (73.4%)	5.90%	3.774	0.052
2.76-17 (Normal)	125 (52.5%)	113 (47.5%)	94.10%		
Total	129 (50.9%)	124 (49.1%)	253		

**Table 7:** Linear regression analysis of hemoglobin levels

**Predictor**	**B**	**Std. Error**	**Beta**	**t**	**p value**	**95% CI**
(Constant)	12.935	0.613		21.096	0	11.727 to 14.143
Iron	0.012	0.002	0.337	5.118	<0.001	0.007 to 0.016
Transferrin	0	0.002	0.024	0.301	0.764	-0.003 to 0.004
TIBC	-0.003	0.001	-0.182	-2.35	0.02	-0.005 to 0.000

**Table 8:** Consumption of folate-rich foods among adolescents

**Dietary Component**	**Consumed (Boys, n)**	**Consumed (Girls, n)**	**Total (n)**	**Percentage (%)**
Leafy Vegetables	122	118	240	94.90%
Fruits	123	113	236	93.30%
Pulses/Sprouts	93	73	166	65.60%
